# Rare diseases and space health: optimizing synergies from scientific questions to care

**DOI:** 10.1038/s41526-022-00224-5

**Published:** 2022-12-22

**Authors:** Maria Puscas, Gabrielle Martineau, Gurjot Bhella, Penelope E. Bonnen, Phil Carr, Robyn Lim, John Mitchell, Matthew Osmond, Emmanuel Urquieta, Jaime Flamenbaum, Giuseppe Iaria, Yann Joly, Étienne Richer, Joan Saary, David Saint-Jacques, Nicole Buckley, Etienne Low-Decarie

**Affiliations:** 1 Astronauts, Life Sciences and Space Medicine Canadian Space Agency, Government of Canada, Longueil, Canada; 2 Molecular and Human Genetics, Baylor College of Medicine, Houston, TX USA; 3 The Strategic Review Group Inc., Ottawa, Canada; 4 Legislative and Regulatory Modernization, Health Canada, Ottawa, Canada; 5 Pediatric Endocrinology and Biochemical Genetics, Montreal Children’s Hospital-McGill University, Human Genetics and Pediatrics, McGill University, Montreal, Canada; 6 Children’s Hospital of Eastern Ontario Research Institute, University of Ottawa, Ottawa, Ontario Canada; 7 Translational Research Institute for Space Health (TRISH) and Department of Emergency Medicine and Center for Space Medicine, Baylor College of Medicine, Houston, TX USA; 8 Canadian Institutes of Health Research Ethics Office, Ottawa, Canada; 9 Department of Psychology, Hotchkiss Brain Institute, and Alberta Children’s Hospital Research Institute, University of Calgary, Calgary, Canada; 10 Centre of Genomics and Policy, Faculty of Medicine, Human Genetics, McGill University, Montreal, Canada; 11 Canadian Institutes of Health Research Institute of Genetics, Ottawa, Canada; 12 Department of Medicine, Division of Occupational Medicine, University of Toronto, Toronto, Canada; 13 Present Address: The School of Health Sciences, University of Western Ontario, London, Canada; 14 Present Address: Hawaii Institute of Marine Biology (HIMB), Kaneohe, HI USA; 15 Present Address: University of Waterloo, Waterloo, Canada; 16 Present Address: Astronauts, Life Sciences and Space Medicine Canadian Space Agency, Government of Canada, Longueil, Canada; 17 Present Address: Directorate of Human Spaceflight and Robotic Exploration, European Space Agency, Noordwijk, Holland; 18 Present Address: Agriculture and Agri-Food Canada, Government of Canada, Montreal, Canada

**Keywords:** Occupational health, Diseases, Medical research

## Abstract

Knowledge transfer among research disciplines can lead to substantial research progress. At first glance, astronaut health and rare diseases may be seen as having little common ground for such an exchange. However, deleterious health conditions linked to human space exploration may well be considered as a narrow sub-category of rare diseases. Here, we compare and contrast research and healthcare in the contexts of rare diseases and space health and identify common barriers and avenues of improvement. The prevalent genetic basis of most rare disorders contrasts sharply with the occupational considerations required to sustain human health in space. Nevertheless small sample sizes and large knowledge gaps in natural history are examples of the parallel challenges for research and clinical care in the context of both rare diseases and space health. The two areas also face the simultaneous challenges of evidence scarcity and the pressure to deliver therapeutic solutions, mandating expeditious translation of research knowledge into clinical care. Sharing best practices between these fields, including increasing participant involvement in all stages of research and ethical sharing of standardized data, has the potential to contribute to humankind’s efforts to explore ever further into space while caring for people on Earth in a more inclusive fashion.

## Introduction

**Fig. 1:**
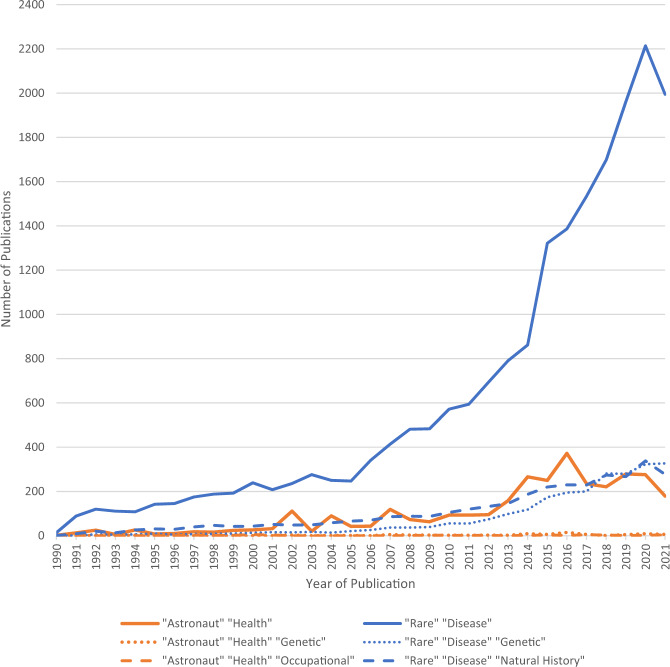
Number of publications for rare disease and space health through time. Rare disease, despite being individually rare collectively affect a significant proportion of the population and thus elicit a higher research output than space health. Even with the revolutions in genomics, genetic studies in astronauts remain rare. Despite commonality of genetic basis for rare disease, genetic studies have increased in the field of rare diseases but they do not represent a dominant research topic in the scientific literature nor even, surprisingly, a growing proportion of the scientific literature. Consideration of “natural history” seems mostly absent from the space health literature but appears as often as genetic in the rare disease literature. The number of annual publications for the keywords “rare disease” (blue) and “astronaut” + “health” (orange) from 1990 to present day (2021). Data was additionally generated for keyword combinations such as “rare disease” and “natural history” (green), “genetic” and “astronaut” + “health” (yellow), and “genetic” and “rare disease” (red). No publications were found for the “astronaut” + “health” and “natural history”. All data was generated using Web of Science. Data in Source data 1.

**Fig. 2:**
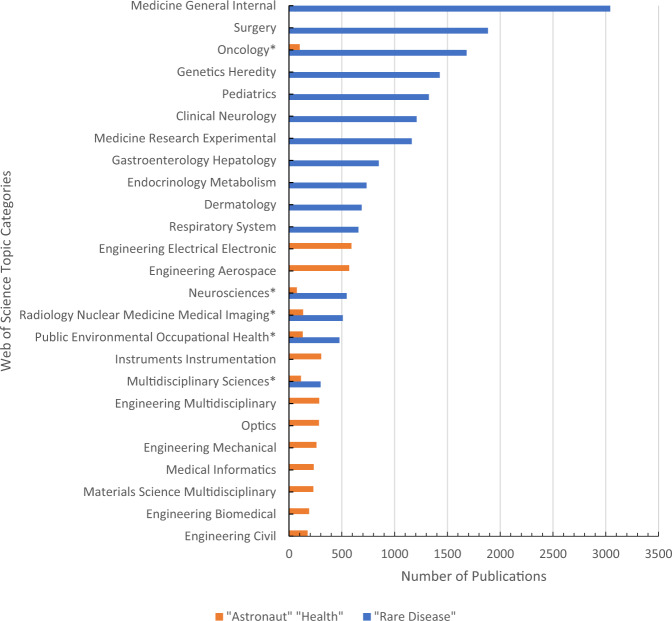
Number of publications for rare diseases and space health as a function of topic categories. Despite a common focus on health, the fields of rare diseases and space health have little overlap in terms of dominant research topics. Space health appears to be technology or engineering oriented, while rare disease integrates a more medical focus. The top 26 most common research categories based on number of publications were generated from Web of Science for the keyword’s “astronaut” + “health” and “rare disease”. The top 10 categories based on publications were graphed for both “astronaut health” and “rare disease”. Five out of 52 total research topics found in common between both keywords were also graphed and denoted with * (Multidisciplinary Sciences, Oncology, Neurosciences Public Environmental Occupation, and Radiology Nuclear Medicine). Data in Source data 2.

For tackling complex issues, the value of bridging across disciplines is recognized for addressing scientific questions of pressing societal significance^1^. As such, domains that share more commonalities may advance faster than disparate areas^1^. Rare disease and space health are two health domains for which interdisciplinary collaboration may appear challenging, as they are at first glance fairly disparate, whether spatially (terrestrial vs. celestial environments), etiologically, demographically, methodologically, or ethically (Table 1). With just around 600 people having reached Earth orbit, astronaut health in space could be considered a niche research subject^2^. While this number increases significantly when taking into account participants in ground analogs, it can be difficult to accurately mimic the physiological and psychological responses to spaceflight. In contrast, rare diseases, though individually rare, are estimated to affect upwards of 300 million people across the thousands of known rare diseases^3^. This is reflected in the research output in terms of the number of scientific publications referencing rare diseases which is proportionally larger than for space health (Fig. 1). Whereas rare diseases are predominately characterized as permanent genetic disorders, conditions linked to space exploration are often transient results of exposure to occupational health hazards among predominantly healthy adults^4,5^. These differences lead to variation in the approaches to research and therapeutic care, between rare diseases and space health and even to the process of how research topics are selected and how knowledge is converted into health solutions. This contrast is reflected in the coverage of genetic and occupational consideration in the linked scientific literature (Figs. 1 and 2). Despite striking differences, there are clear avenues for exchange between rare disease and space health research and care.

**Table 1:** Comparison of key descriptive characteristics pertaining to rare diseases and space health.

Defining attributes	Rare disease	Space health
Definition	A disease that affects a small proportion of the population	A branch of research dedicated to supporting human physiological, biological, and psychological health during and after space flight
Sample sizes	Europe: <1 in 2000^15^; US: 200,000 at a time (total)^15^; Canada: <5 in 10,000 (proposed)^79^; Australia: <2000 people (total)^79^ However, rare diseases in aggregate affect 300 million people worldwide^3^	^a^Reached the altitude of space (FAI definition): 596^2,36^ Reached the altitude of space (USAF definition): 609^2,36^ Reached Earth Orbit: 579^2,36^
Sample size composition	69.9% of rare diseases have pediatric onset^31^ Certain population groups may be more at risk for some rare diseases than others (i.e., Ashkenazi Jewish population and Tay-Sachs)^15^	89% of space travellers were male^36^ 11% of space travellers were female^36^ Demographics are becoming more diverse—The 2020 NASA and CSA class of astronauts included 6 women (5 NASA, 1 CSA) and 7 men (6 NASA, 1 CSA) with 5 of the astronauts being people of color (5 NASA)^80^. Civilian commercial spaceflight will likely increase the diversity and number of samples.
Alternative trial designs	^a^Randomized-control trial SMARTs ^a^N-of-1 ^a^Case-control	^a^Randomized-control trial ^a^N-of-1 ^a^Case-control
Genetic testing	Yes—71.9% of rare diseases have a genetic basis^31^	Genetic testing currently precluded from screening measures
Countermeasures	No	Yes (exercise, medication, etc.)
Resource funding	Non-profit, government funding, private investors, research grants	Primarily national space agencies

United States Air Force definition of 80.6879 km (12 miles below the FAI definition)^81^.

Data updated as of December 13, 2021.

^a^Fédération Aéronautique Internationale (FAI) definition of the Karman line which is 100 km above Earth’s average sea level.

There are examples of valuable knowledge transfers between the fields of rare diseases and space health. Overlaps exist in terms of terrestrial prevention strategies, as well as underlying physiological explanations. For example, investigators have unveiled a genetic predisposition for astronauts to develop ophthalmologic issues such as choroidal folds, lines in the posterior pole of the eye, similar to women suffering from polycystic ovary syndrome (PCOS)^6^. Initial studies have shown an association of one-carbon metabolism pathway polymorphisms with ophtalmic changes^7,8^, which could lead to potential terrestrial prevention strategies^7^. From a neurovestibular perspective, the changes in gravity fields may also cause a decline in the ability of astronauts to orient in the surrounding^9^, a phenomenon similar to the one experienced by individuals affected by a rare familial disease^10^ known as developmental topographical disorientation (DTD)^11,12^.

Commonalities between rare diseases and space health are not limited to specific overlaps in physiological explanations for phenomena. Notably, both areas are characterized by a lack of comprehensive, diverse, and validated personal data in order to derive novel scientific solutions. Moreover, rare disease and space health research often entail small sample sizes that influence the types of trials that can be used and, subsequently, the methods of data analysis adopted^13^. Indeed, some types of research trial designs are ethically and statistically inappropriate in these circumstances. General universal ethical principles mandate that with a paucity of participants in both fields, it is crucial to ensure that participants are consulted about the choice of available research topics, their priorities, and therapeutic options in order to align participants’ and researchers’ priorities to research outcomes. In order to minimize some of the identified barriers to conducting research in a rare disease or space health context, increasing collaborative data sharing and open model research is lauded for potentially establishing a foundation for future experimentation^14^.

To better guide our evaluation and discussion, we conducted a baseline literature search to identify synergies and discrepancies between the 2 areas of research (rare disease and astronaut health). We searched PubMed using the keywords “astronaut” “health” and separately “rare disease” from the years 2000–2021. In order to have the most similar sample sizes for both searches, all “astronaut” “health” articles were included, while “rare disease” articles were sorted by “Best Match” in descending order, and the top 5 original research articles for each year (2000–2021) were included. Articles were then reviewed and original articles were included per the protocol outlined in the Supplementary Materials, which includes the data collected. The results from the PubMed search are referenced in text. Bibliometric data for Figs. 1 and 2 used in figures was obtained separately from Web of Knowledge, which provides category information. Data is provided in Source data 1, 2, and 3 and further details about data collection are provided in Supplementary Methods.

Here, in an effort to identify areas of translational opportunity where one area could benefit from the other, or in which both fields could derive benefit, we first expand on the definition of rare disease and space health and elaborate on how defining differences could lead to contrasts in the selection of research topics to be investigated. Then, we delve into shared challenges found across rare disease and space health areas (i.e., limited long-term knowledge and small sample sizes) and highlight the opportunity of implementing collaborative data-centric strategies, as well as individualized approaches in both research and care.

## Definitions and participants at hand

Definitions of what constitutes a rare disease depend on the incidence rate of the specific disorder in the population. These rates often coincide with the pharmaceutical regulations surrounding orphan drugs (drugs for rare diseases) and vary from country to country. In the United States, rare diseases have been defined as affecting 200,000 individuals or less while Canada has proposed to define it as <5 in 10,000^15,16^. Out of over 7,000 rare diseases, 72% are linked to a genetic condition and 70% primarily affect children^17^. Given how many rare conditions are inherited and the permanent nature of those conditions, a substantial proportion of rare disease research aims to develop effective therapeutics by investigating the genetic targets/pathways of the disease (Fig. 1). Health conditions linked to human space exploration may well be considered as a narrow sub-category of rare diseases, as these conditions only affect a subset of the already small population of astronauts. However, in contrast to the majority of rare disease, conditions linked to human space exploration are seen as resulting from occupational health hazards present in an environment in which generally healthy, highly-screened adults have decided to take certain calculated risks, generally, in the context of employment. This etiology is reflected in the space flight-associated scientific literature in terms of reference to occupational considerations (Fig. 1). More recently less screened civilians flying with commercial spaceflight companies have also had increasing access to the space environment. Evidence reviewed by NASA through the Human Research Program (HRP) has categorized the risks of space exploration missions into the categories of Human Factors and Behavioral Health Performance, Space Radiation, Exercise and Extravehicular Activity, and Exploration Medical Capabilities^18^. Similar categories for space biomedical research are adopted by the Canadian Space Agency (CSA), Japan Aerospace Exploration Agency (JAXA), and the European Space Agency (ESA) to guide research topics^19–21^.

Both rare disease and space health research and care are hindered by the deficiency of diversity or representativeness of the people who engage with the research as subjects and the wider communities affected. In the rare disease context, there is high heterogeneity in both phenotypic expression of rare diseases and treatment effects, representing a practical challenge to measuring the success of clinical treatments^22,23^. Despite this, the genomic information available in rare disease databases overwhelmingly comes from individuals with European ancestry with other ancestries underrepresented in population studies^24^. A lack of comprehensive genomic data can make it more challenging to differentiate between a ‘rare’ disease and a condition that is more common amongst individuals that are not of European ancestry. Astronaut cohorts have historically been mainly homogeneous and typically consisting of middle-aged men of European descent who maintain exceptional physical fitness and fit within a certain height range. While physical fitness remains a requirement for NASA and International Partner astronauts, there has been an increasingly diverse population of flyers including more women and those of non-European descent that will continue to grow with the possibility of more accessible commercial spaceflight opportunities^25^. Certain conditions pertaining to space flight also impact individuals differently based on sex, including references to how women have greater loss of blood plasma volume than men during spaceflight and women’s stress response includes heart rate increase while men respond with increase in vascular resistance. Similarly, race, ethnic groups, and sex can have varying space radiation cancer risk predictions, with Asian-Pacific Islanders and Hispanic populations having the lowest overall cancer risks, and White females having the highest^26^.

Differences in the etiology of conditions associated with space health and rare diseases lead to differences in care. Health research conducted in space has been focused on characterizing the physiological responses to living in the extremes of space and on prevention of those deleterious to crew health and performance^18^. Space agencies have implemented preventive measures (countermeasures) to ensure that astronauts maintain a good health during and after space missions, and to reduce the impact of known space-related physiological adaptive processes such as loss of bone density and muscle and changes in neurovestibular function^27^. In juxtaposition, rare disease clinical care is primarily focused on diagnostic treatments and therapeutics that rely on both valid applicable evidence in tandem with clinical expertise^28^. With greater recognition of environmental influences on the onset and phenotypic presentation of rare disorders, increased population genetic screening may reveal that there are identifiable preventative measures such as avoiding exposure to toxins and lifestyle changes that one can take to reduce the onset of certain disorders^29^.

## Selection of research topics

Likely due to the contrast in etiology and focus of care, rare disease and space health fields identify and prioritize research questions differently (Fig. 2). Rare disease research appears to have a researcher-led nation-agnostic development of questions, which is common to most life science fields of research. While space health research has a more centrally-guided research mandate put forward by national space agencies based on solving issues that could impact astronaut health, and that would negatively impacting lengthy and costly space missions.

In addition to the government programs that commonly fund health research, large foundations such as the National Organization for Rare Disorders (NORD) and the Rare Disease Foundation as well as many disease-specific not-for-profits and charities, also fund rare disease research^30,31^. Some efforts are made by the rare disease research communities and these foundations, to identify common questions and ways forward^32^. Of the articles reviewed, 8% of rare disease articles reported receiving funding from a foundation. The rare disease research community has pioneered the involvement of patients and their families at the very onset of research to identify worthy research topics and ensure that research priorities align with what is considered clinically important to the family or the patient. In light of many rare disease patients having shortened lifespans and limited treatment options, patient involvement in research and trial design greatly enhances a sense of self-efficacy and the ultimate quality of care provided^33^.

In comparison, space health research questions are generally driven by the mission risks and the engineering mitigation measures to alleviate those risks as identified by national space agencies (Fig. 2). The reason for this focus is likely in part driven by the source of funding for this research, with 70% of astronaut health articles reported receiving funding from a space agency and 45% received funding from government bodies. In sharp contrast, only 14% of rare disease articles reported funding from government bodies and topics were generally medical. Space health research may benefit from a diversification of sources of funding, including more generalist health or science funding agencies, and from research that goes beyond evaluating engineering solutions to health risks to greater integration of topics common in rare disease research, particularly genetic heredity. On the other hand, rare disease research may find value in engaging engineering solutions to mitigate the triggers or effects of rare diseases. Also, we have preferred the use of the term space health, where others may have used space medicine, to reflect that the focus of most of this work is not medical in nature.

As of November 2020, 3000 experiments had been conducted onboard the International Space Station (ISS), mostly by astronauts, with 300 of these experiments involving human research. This participation highlights the high degree of skill among astronauts required to perform innovative life science experiments, among the dominating physical and material sciences experiments^34^. Despite their involvement in the execution of research experiments, astronauts are not always involved in the process of generating research questions and designing experimental protocol to better tailor the space health focus on both their needs and interests^35^. Increased astronaut involvement in research, mainly through greater stakeholder power in determining experiments (i.e., ensure research addresses what matters to the astronaut not only to the researcher) could increase the relevance and uptake of the research outcomes. Both rare disease and space health communities could achieve greater participant satisfaction by prioritizing participant involvement throughout the research process and ensuring a ‘real’ partnership with participants prior to enrollment in research clinical trials.

## The sample size issue: how small is small?

Research involving distinct populations, such as rare disease patients and astronauts, are associated with small sample sizes. For space health, approximately 600 humans have travelled to space, with a limited number of participants available for research this far^36,37,38^. Moreover, the limited number of flight opportunities associated with the high cost of space travel is often cited as a fundamental obstacle to carrying out experiments in the near-Earth orbit^39^. While hundreds of millions of people worldwide are affected by rare diseases, the number of patients developing a particular disease is low compared to other prevalent diseases, and often vary in frequency from less than a dozen documented cases to millions globally^28^. Moreover, as for any disease and treatment, genotypic and demographic variability within rare diseases further reducing the population that can be targeted with a given therapy, as patients may have vastly different responses to treatments^40^. Of the articles we reviewed, the median [interquartile range] study sample size for astronaut health was 13 [7–26] participants while the median rare disease sample size was 1 [1–16]. Despite the low incidence rate of rare diseases, cross-national collaboration and extensive database information can allow rare disease researchers to obtain larger sample sizes. The target size (number of study participants that are either people with rare diseases or astronauts) in the articles we reviewed was significantly lower, with astronaut health having a median of 1 [0–12] participants and rare disease with 1 [1–9.5]. The requirement for substantial sample sizes impacts the study design of prospective research trials.

Typically, when it comes to choosing a design to test the effectiveness of an intervention, randomized-control trials (RCTs) have long been regarded as the gold standard to producing reliable evidence. However, rare disease researchers have found that RCTs have questionable reliability in rare disease research, largely in part due to small sample sizes^41^. When trial designs do not contain a sufficient sample size and statistical power, alternative designs and analyses can allow research to proceed on the grounds that the research question has great clinical significance^13^. Bayesian approaches can permit the incorporation of real-time knowledge into the ongoing clinical trial and analysis. This provides an opportunity for rare disease researchers to pivot when encountering novel information and to enhance the trial design as opposed to starting from scratch. Such approaches include adaptive design that makes use of ongoing trial data to modify trial design aspects as need be^42^. Proposed designs include the use of sequential multiple assignment randomized trials (SMARTs) that can allow comprehensive testing of the efficacy of multiple drugs for a particular rare disease to determine which drug can best serve as the standard therapy for a particular disorder^43^. Moreover, implementation of “N-of-1” trials in which an individual participant undergoes consecutive periods of treatment(s) or placebo, have been published in both the rare disease and space health literature^41,44^. In the articles reviewed, we found that 63% of the rare disease articles were case studies (*n* = 1) while only 5% of the astronaut health studies were classified as case studies. For both rare disease and space health, experimental design and statistical approaches are likely to continue to evolve to better address the challenges of small populations, with the need for regulation to follow.

One of the main barriers to conducting clinical trials in the setting of orphan drugs for rare diseases is the recruitment of patients. To combat low patient recruitment, involving rare disease patients and astronauts in the experimental design process has been proposed^13^. Involving research participants in the experimental design has great potential, however, the burden (i.e., time, travel, and financial considerations) associated with participation may prove to strain the development of research, especially in communities that are seeking larger sample sizes^45^. Longitudinal, extensive follow-up evaluations commonly associated with rare disease trials can deter otherwise willing participants and families from participation.

In order to overcome this challenge, it has been suggested that clinical trials be carried out with multicenter involvement instead of using one facility to conduct all research experiments^46^. Of the articles reviewed, 29% of astronaut health studies involved a multicenter layout, while only 11% of rare disease articles were designated as multicenter. To continue to do so, technological advances can be used to stimulate patient participation through gathering sporadic and continuous patient information from home through modern methods of data capture^47^. These advancements can encourage patient registration in research, especially for families and patients that are reluctant to participate due to financial and psychological concerns over long-term travel and follow-up. Similarly to rare diseases, astronaut participation in research is voluntary, however, due to the occupational nature of the health concerns being addressed, ongoing surveillance of occupational health hazards may serve as a requirement for employment^48^.

In an attempt to alleviate the burden of research participation placed on astronauts, alternative environments, such as analog environments and simulation facilities, have been proposed^18,49^. While there are limitations to using ground-based analogs, this often remains the most feasible and viable alternative to best prepare for space exploration-class missions and obtain relevant data when space travel is not a possibility^18^. For space health, microgravity analogs such as parabolic flight have been used. Facilities with “analog” relevance for rare diseases include organ-on-a-chip micro-scale systems that are designed to simulate human tissues can be instrumental in advancing rare disease research, especially when intervention therapy is not a possibility^49^. Utilizing analog approaches from space health research may prove to be instrumental in rare disease research, especially concerning rare diseases with short lifespans and ones where unstable patient conditions do not allow for human research to be performed.

Small sample size research demands a methodological high standard for data analyses^14^. Rare disease researchers must in turn be able to produce statistically significant results that adhere to regulator standards in order to use the data to get approval for therapeutics^42^. Moreover, the lack of replicability of current space health research poses a significant problem in the area^50^. For both communities, it can be concluded that a small sample size highlights the limitations of applying traditional statistical methods to conduct research and is the major challenge for the need to generate evidence and find curative treatments.

Given the challenges implied in meeting statistical requirements, many have raised the issue of relaxing standard margins for statistical significance in regard to designing clinical trials, pending careful considerations of the cost/benefits for all stakeholders^42^. As an alternative, authors suggest that the main criteria for publication should revolve around the pertinence of the study in adding knowledge of clinical or public health, as well as on the validity of the methodology and the experimental rigor of the study design. The US Food and Drug Administration (FDA)’s popularly used Guidance for Industry on Enrichment Strategies for Clinical Trial to Support Approval Of Human Drugs And Biological Products outlines flexible evidence standards to show drug efficacy for low-frequency molecular alterations by using those “mutations” to identify patients with specific biomarkers and patients with a greater chance of prominent worsening conditions^51^. This is part of the FDA’s efforts to advance the development and availability of effective treatments for rare diseases^40^. To atone for the lower empirical standard, the FDA recommends detailed, transparent labeling information for drugs, especially pertaining to information about the level of evidence supporting the therapeutic^40,51^. Consequently, this change could assess gaps in knowledge and stimulate beneficial collaborations between scientists to share and combine data to increase sample size.

## Natural history as a mitigating factor to small sample size and knowledge gaps

Numerous challenges to rare disease research and therapeutic development include: (1) relatively rare viable and tailored treatments or approved therapies, (2) uncertainties in diagnostic detection and (3) in establishing robust endpoints, and (4) the existence of large knowledge gaps in natural history^42,50^. Defining the right variables and parameters for rare disease clinical trials is especially challenging, since the understanding of underlying mechanisms and conditions for disease development and treatment remain poorly understood^52^. Research in space health faces similar issues as the high costs of space missions, methodological constraints and specifics associated with conducting research in an isolated, confined, and extreme (ICE) environment, complicating the mobilization of knowledge into action^39^. Amongst other challenges in the design of space health experiments, the high cost and difficulty associated with transporting equipment into space, as well as the necessity to develop protocols that are tailored to the ICE environment are often regarded as contributing factors to the lack of consistent information in the field. Rare disease and space health fields are often tasked with experiments that cannot be easily reproduced, which is problematic, as reproducibility (along with predictability and falsifiability) are the cornerstone principles of experimental research.

Space health research often focuses on countermeasure development, which can be insufficient in addressing all space health risks, as seen by the limited progress in areas such as risk of cancer caused by HZE, high atomic number and high energy, charged particle radiation^4^. In order to achieve better astronaut health and performance mitigation strategies, promoting and providing high-value applied research concerning the efficiency of treatments and consideration of novel treatments should be taken into consideration^18^.

The scarcity of individuals affected by individual rare diseases and of astronauts who have travelled to space, as well as the heterogeneity of those individuals, has contributed to the knowledge gaps in both fields (Fig. 1). As few as 70 articles include data collected during space flight addressing psychological, behavioral challenges and performance of astronauts arising from space exposure^38^. Improving natural history studies has been proposed to address small n sample sizes and replication concerns, thus helping refine questions and fill knowledge gaps in space health and rare disease research^53^. A natural history study collects health information over time to understand how the medical condition or disease develops and to give insight into how it might be treated^53^. The study of natural history can provide a foundation for informing future treatments, biomarker identification, and facilitating the translation of research into therapy^54^. The long-term diagnosis associated with the majority of rare diseases requires in-depth knowledge on biological mechanisms at every stage in disorder progression, thus natural history studies play a prominent role in identifying gaps in existing scientific knowledge^55^.

Comprehensive natural histories, particularly before or long after space flight, have been notably rare in this field (Fig. 1), however, long-term monitoring of astronauts has been used for decades to better inform research questions. Regardless of spaceflight experience or time in the astronaut corps, long-term health data has been captured and is at least theoretically available for research pending consent for all NASA astronauts (*n* = 360) (information valid as of August 5, 2022 provided by the NASA Lifetime Surveillance of Astronaut Health -LSAH-). These long-term monitoring initiatives are akin to natural history studies for rare diseases, but would benefit through greater international implementation. Along those lines, the integration of research evidence suggests that, in order to achieve better astronaut health and performance mitigation strategies, performing a constant monitoring of astronaut’s health, validating the current astronaut selection process and refining and improving the selection system should be taken into consideration^18^.

## Tailored approaches

As more scientific knowledge is being harnessed in both areas of research, we now understand the relevance of more tailored approach to research and care, factoring in spatial, genetic, and environmental heterogeneity for rare disease and space health research participants. New rare diseases are being discovered or characterized annually. As approaches to research and care need to be adapted to individual rare disease^56^, new approaches need to be continuously developed^56^. Heterogeneity (including in terms root cause, symptom presentation, therapeutic course, and response to treatment) within and among rare diseases can contribute to the difficulties associated with the efficacy of treatments. While genetic variation is high among various rare diseases, genotypic and phenotypic divergence exists even within individuals affected by the same rare disease. For rare diseases, the variation that is addressed is primarily physical differences between individuals and their clinical condition, though consideration of environmental factors is beneficial. Tailoring approaches in the context of space health requires consideration of variations between individuals, but also differences in mission variables (dose exposures, mission length, destination, and occurrence) and the interaction between these two factors^18^.

Next-generation sequencing (NGS) has been an important factor in increasing the capacity for identifying the genetic basis for rare diseases, allowing people to receive tailored research and care, that takes into consideration genetic variations that distinguish rare diseases and variations within specific rare diseases^57^. Space health research and care does not appear to have benefitted to the same extent from advancements in genomics, potentially because of the pervasive issue of genetic data regulations for employment when it comes to research and development involving human genetics^58^. It is NASA’s policy to only voluntarily obtain and use human research genetic testing for risk identification related to space exploration and informing clinical care^59^. The responsibility to protect the privacy of astronaut genetic information to the fullest extent of the law as per GINA (the Genetic Information and Non-discrimination Act) is outlined in NASAs Policy Directive and prohibits the use of human genetic information for employment decisions related to astronaut selection, training, and missions^59^.

However, the European Space Agency recently found that the individual response to approximately one-third of drugs available on the International Space Station are substantially affected by heritable polymorphic metabolizing enzymes^60^. This shows how standardized screening and testing may have significant benefit for tailoring astronaut countermeasure regimes to reflect individual need. The field of space health research could reap benefits of adopting genetic screening approaches from the rare disease field to improve clinical outcomes. Future technological advancements in the space exploration field will allow for the production of therapeutic molecules tailored to the needs of a patient, potentially implemented aboard spaceships for long-duration cosmic missions. Such adapted approaches include experimental designs that align treatments to specific subgroups in a larger sample size, allowing for a generally more efficient allocation of health resources.

In the context of rare diseases, since treatment options are often uncertain and are greatly influenced by patients and their families’ preferences regarding treatment avenues, incorporating patients as active decision-makers can reveal important considerations for trial designs^33,45^. Furthermore, patients can provide invaluable insight into treatment effectiveness and potential side-effects, allowing researchers to optimize both existing and future therapeutics. Taking into consideration the variety of physiological responses/symptoms manifesting in rare diseases and astronaut health, personalized approaches can more effectively utilize existing treatment regimens and tailor them to specific individuals/groups.

Personalized clinical care brings a new outlook to the table which includes predictive and tailored therapeutics, earlier interventions and better clinical endpoints, and hence greater general effectiveness^23^. Integrating the evaluation of various patient characteristics and interactive feedback between patients and clinicians throughout the entire process of clinical trials could be of highest value to define tailored clinical outcomes and to enhance our understanding of the most suitable treatment administration for both patients and clinicians^22^. Moreover, a recent review study suggested that the enrollment of participants in clinical trials as decision-makers as opposed to solely study volunteers can optimize interpretation of clinical outcomes^61^. Personalized approaches, whether in the form of dynamic variables or tailored care, represent an area for space health and rare disease fields to invest in.

## Collaborative data management

Due to the complex issues faced in rare disease and space health research, stakeholders have much to gain through comparable approaches to data management. After experiments are conducted, great pressure is placed on ensuring the viability of the samples and subsequent data analysis procedures. The collection, processing, and storage of resources and samples associated with space health and rare disease research require comprehensive data standardization processes. Moreover, compiling global or multinational standards to how evidence is collected while increasing data sharing among communities can mitigate the reproducibility and validity concerns associated with rare disease and space health studies^46^. The design of the Core Outcome Measures in Effectiveness Trials (COMET), while not specific to either field, could serve as a crucial tool in establishing a minimum foundation of outcomes to be included in future trials around the world^62^. Global initiatives that bring experts in their respective communities together can be employed to maximize the use of generated scientific data while taking into consideration the limited resources available to both communities^14,35^. That being said, establishing international partnerships in rare diseases and space health research and development represent an additional challenge, given the plurality and heterogeneity of participants encountered in those fields.

Promoting international efforts to create standardized regulatory and ethical data governance policies would be highly beneficial for rare diseases research and development. Such policies would promote collaborative research and thus prevent knowledge duplication. Fostering collaborations between countries can also lower the expenses of translational research for many stakeholders, and guarantee an easier, faster access to therapeutics to patients^23^.

Similarly, for the space health community, collaboration between individuals and research groups is truly important^63^. The fact that the ISS is an international research facility, which is also the main platform available to scientists to gather space health in-flight data, ensures collaborations between various space agencies. As ISS partner nations conduct their research programs, international collaboration and exchange among scientists worldwide is growing rapidly. The many research projects based onboard the ISS are often the results of cooperation between many ISS partners. Japan (JAXA) and Russia (Roscosmos) teamed up to study new treatment options for Duchenne Muscular Dystrophy through a protein crystal growth experiment in 2009, providing insights into potential biological pathway targets for treatment, which underpins the value of multinational collaborations in the investigation of novel treatment avenues^64^. Moreover, NASA reiterates that such partnerships initiated between research groups are key elements to promote and instill collaboration and teamwork values amongst stakeholders and to work toward efficient problem solving^65^. Recently, the exponential growth in civilian commercial spaceflight will bring new opportunities to collect more diverse data in a high throughput fashion.

In parallel, there is evidence of a collective effort and interest toward data standardization in the rare diseases’ community as the case of Myotonic Dystrophy (i.e., DM), where health care professionals took part in an international collaboration initiative that is the D-M Scope patient registries^66^. In the rare disease community, the increased use of extensive databases such as Orphanet, serves as a model for international data collection^67^. As a further example, The Matchmaker Exchange (MME) has shown how international data sharing in the rare disease realm can be optimized by enabling searches of multiple databases at once, while allowing for quicker identification of rare genotypes and phenotypes in a manner respectful of participants confidentiality right^68^. Matching algorithms of the MME have shown promising success in rare disease gene discovery by using participant(s) genotype and phenotypic features to retrieve similar cases^69–72^. The implementation of core outcomes for rare disease registries, as seen in COMET, can further standardize existing databases, and eliminate fundamental discrepancies among and/or within registries.

In order to maximize the benefits associated with data exchange between researchers, establishing a common language for data standardization is crucial to ensuring data is easily and accurately interpreted. Data standardization comes about through different implemented approaches. In space health, researchers have resorted to the lowest common denominator approach as a means to define the variables contained in the omics datasets^73^. Rare disease and space health researchers must work with a finite number of resources; therefore, quality data collection standards are essential to preserving quality evidence.

Over the past years, NASA’s Life Sciences Data Archive (LSDA), PubSpace, NASA NTRS as well as GeneLab initiatives, have sought to improve data availability and thus can be regarded as a great leap forward in working with sensitive data^74,75^. Along those lines, a recent review revisiting the implications of open model research suggested that NASA’s open innovation research model, involving open peer-production, fostering collaboration amongst research, and development professionals, has spurred the development of scientific knowledge^76^. Of the articles reviewed, 29% of the astronaut health articles reported using databases in their research and 16% of the articles had open access availability.

Primarily given the wide array of rare diseases and low incidence rate, it is highly unlikely for a single group to advance research alone^77^. Data sharing may be a valuable resource in understanding natural history, disease progression, and providing an adequate sample size to work with. Of the articles reviewed, only 8% of rare disease articles used public databases. Given that pathophysiology occurring in astronauts can be characterized as occupational-based, crew members are not able to opt out of occupational surveillance as it is intended for use only within the organization to better understand the hazards associated with spaceflight^18^. While participation in research is voluntary, space organizations are aware of the potential for coercion and thus have rigorous informed consent procedures^48^. Due to the small number of individuals available for research, international data sharing can lead to direct identification of participants, even with privacy regulations in place. Individuals with rare diseases who seek support through patient support forums found on rare disease foundation sites or webpages are at high risk of re-identification in hospital datasets due to their unique identifier combinations (e.g., age, sex, rare disease, marital status)^78^. Astronauts face additional privacy concerns about identifiability due to their visibility in the public eye.

In the context of rare diseases, participant enrollment in clinical trials, is driven by the physical, financial, and emotional burden and day-to-day impacts of rare diseases on the patient and their communities^45^. A sense of commitment to the research cause is present in participants from both scientific fields, however, for many rare disease patients this is often an urgent effort to discover new possible quality of life-preserving treatments. Ensuring that rare disease patients and their families receive in-depth information, are supported prior to enrollment, and are able to give and revoke consent for participation at any point is crucial to successful and non-exploitative research.

Data privacy laws are increasing in sophistication globally to allow for a transition into the big data era of mass data sharing, often resulting in tightened laws with higher consent standards and safeguards in place, allowing for less flexibility to share samples and data internationally^77^. In order to achieve a balance between too lenient and too rigid privacy laws, increased patient involvement in research design, security protections and transparency about how data sharing occurs have been recommended^48,77^. The extent of the experimental information collected and the degree of privacy that’s regulated will remain a topic that requires further discussion among stakeholders in both communities.

## Summary and future outlook

As humankind strives to explore ever-further into space while caring for people on Earth in a more comprehensive and individualized fashion, we will need to continue to enhance our approaches to both science and healthcare. Monitoring the physiological and psychological effects of space in a manner similar to what is done for tracking the life history of a rare disease may provide unique insights into health outcomes for astronauts. Across all research and care, ensuring that the people most directly affected are enabled to partake and that they have their voices heard throughout the process is crucial. This participation of astronauts or an individuals affected by rare diseases both empowers these people and improves the outcomes of research and care. Important advancements in digital technologies will enable the sharing of precious data in ways that increase reproducibility and reuse. This transformation should at the same time be leveraged to offer greater protection of individuals’ autonomy and privacy rights. Specifically, these improvements may change the approaches to astronaut personal information, including genetic data. Research in this area may be enabled and astronauts may receive information on individual level risks and better-tailored mitigations to spaceflight stressors in ways that does not compromise their choices and privacy. Future initiatives in space health and rare disease areas should involve outlining a clear path forward, with area-specific goals and a timeline by which they hope to be accomplished. By overcoming logistical and practical barriers, the space health and rare disease communities may catalyze wider changes in both health research and care.

### Reporting summary

Further information on research design is available in the Nature Research Reporting Summary linked to this article.

## Supplementary information

Supplementary Material

Reporting Summary

## Data Availability

The authors declare that the data supporting the findings of this study are available in the source data files. Source data are provided with this paper.

## Source data

Source data 1
Source data 2
Source data 3
