# Supplementary material for: Rare diseases and space health: optimizing synergies from scientific questions to care
Source: NPJ Microgravity. 2022 Dec 22;8:58. doi: 10.1038/s41526-022-00224-5 (PMC9780351; doi:10.1038/s41526-022-00224-5)
Supplement: Supplementary file 1 — Supplementary Material [file 41526_2022_224_MOESM1_ESM.pdf]

Supplementary Material

Rare Diseases and Space Health: Optimizing synergies from scientific questions to care

Maria Puscas, Gabrielle Martineau, Gurjot Bhella, Penelope E Bonnen, Phil Carr, Robyn Lim, John Mitchell, Matthew Osmond, Emmanuel Urquieta , Jaime Flamenbaum, Giuseppe Iaria, Yann Joly, Étienne Richer, Joan Saary, David Saint-Jacques, Nicole Buckley, Etienne Low-Decarie

Supplementary Methods: Details of data collection including Web of Science (WOS) publication counts for figure 1 and 2 and PubMed Literature Search Protocol

Source data 1: Data for Number of publications for rare disease and space health through time. Used to produce Figure 1.

Source data 2: Data for Number of publications for rare diseases and space health as a function of topic categories for Figure 2.

Source data 3: References arising from the Literature Search Protocol and linked data

Supplementary Methods

1. Figure One

Web of Science (WOS) was used to extract information on the number of annual publications from 1990-2021 for 6 combinations of keywords related to space health and/or rare diseases.
No criteria were put on web of science sources (this included articles, reviews, commentaries etc). On Web of Science, you can include the + or not and it generates the same results and combines both of the words. The way terms were written was how they were researched.
Six seperate searches were conducted for "astronaut" "health", "rare disease", "astronaut" "health" "genetic", "rare disease" "genetic", "rare disease" "natural history", and "astronaut" "health" "occupational".
For each search analyze results was selected, and 'Publication Years' was selected from the drop down list. Data tables were downloaded for all data rows displayed in table but only entries from 1990-2021 were included.
For better visualization data prior to 1990 was excluded as there were only a couple articles available annually for all searches including "astronaut" "health" and this made data visualization difficult.
All values were inputted into excel and visualized as a line graph over the 30 year period of time.

Data found in **Supplementary dataset 1**

2. Figure Two

A search was conducted on Web of Science to see the number of articles in terms of predetermined categories by web of Science.
Two separate searches were conducted using different keywords. The first search used “astronaut” “health” and generated a list of categories that available articles fell under and how many articles are included in each category.
The other search was “rare disease” and also generated a separate list of categories according to WOS. Data tables were downloaded for all categories.
The top 26 most populated categories in each of the two separate searches were included in a seperate excel sheet with their corresponding values.
For Figure 2 the top 10 categories from "rare disease" and "astronaut" "health" as well as the 5 categories that overlapped between both searches were included in the graph.
A total of 25 categories were included and arranged in descending order.

Data found in the **Supplementary dataset 2**

3. PubMed searchArticles 2000-2021 PubMed Literature Search Protocol (in text references to number of publications)

A rudimentary literature search was conducted on Pubmed.ncbi.nlm.gov to guide discussion. Two seperate searches were conducted for articles related to space health and rare diseases.
For articles related to space health the keywords searched were “astronaut” “health”. PubMed reports “astronaut” “health” & “astronaut” + “health” interchangeably. "space" "health" was not used as many articles retrieved were not referring to astronaut health but rather just included the word "space".
In order to receive articles that reference rare diseases one must search “rare disease” as a whole term. When one searched “rare” “disease” or “rare” + “disease” the results were substantially less tailored.
Oftentimes disorders, mechanisms, disease symptoms/manifestations or other characteristics are referred to as rare as opposed to the disease itself and does not fall into what is determined to be a rare disease.
Due to the fact that there were substantially fewer articles available for astronaut health, all articles were included. No filters were included in the initial PubMed search.
For "rare disease" articles were filtered by Best Match and recorded on a mastersheet in descending order. Articles were either included or excluded based on predetermined criteria.
In order to be included, articles must meet ALL of the following criteria: original articles, full text availability, include human subjects, related to the keyword search/topic.
5 articles were included for each year from 2000-2021 for "rare disease".
Articles for both "astronaut" "health" and "rare disease" were retrieved from January 1 2000 to November 16 2021.
For articles that were included the date of publication, case report (YES or NO), sample size, target size, funding categories (Government but not space agency, foundation, space agency, private, no funding or not mentioned), funding detail, duration of study(months), data availability, purpose of study, , multicenter vs unicenter, used database (YES or NO), observational vs experimental, retroactive vs prospective and analysis categories were recorded.
The case report category was to denote whether or not the article was a case study and had a sample size of 1 (n=1 studies). Only Yes or No were recorded.
The sample size was the total amount of participants/subjects included in the study, including the target demographic, control groups and all other subjects.
The target size was how many of the sample size participants were astronauts or people with rare disease(s).
The funding category Gov but not agency refers to funding sources that are sponsored by an organization answering to municipal, provincial/state and/or federal agencies/bodies. This can be organizations such as the NIH or CIHR. The funding category Foundation applies to Foundations and space agency applies to national/public space agencies such as NASA, the CSA or the ESA.
The private funding category applies to private biotechnology, space or health corporations/organizations.
The funding detail category includes the names of all (if any funding categories).
If there was no funding or funding wasn’t mentioned in the article that is denoted on the sheet and “None” is written in the Funding detail category.
The duration of the study (including preliminary tests on subjects and follow up) is listed in months.
Data availability is denoted as Upon request (if the authors or sites must be contacted to receive raw data or models), No indication (if data availability is not mentioned), or Open Access (if the information is available in an open database or in text). One article was denoted as Upon legal basis as data will only be made available if it is legally required.
The purpose of the study revolves around what was the reason for the study and what was accomplished. This includes characterizing physiological symptoms, diagnosis, observing response to

therapeutics/treatment/intervention, clinical trials, characterizing genotype and observing response to environment.

The experiment detailed was more open ended and included any key characteristics related to the experimental design and manuscript. This included if it used an analog facility, if it was a longitudinal study, cohort study, randomized study, repeated measures study, double blind study etc.

Articles were then classified as being either Multicentric or Unicentric. If the study took place at more than one facility or location it was classified as Multicentric, otherwise it was classified as Unicentric.

Articles were classified on whether they used databases by either Yes or No. If no database was mentioned No was written down.

Articles were then classified on whether they were observational or experimental. Studies were classified as experimental if they included controls, an intervention and/or experimental conditions. Studies were classified as observational if they were simply observing a participant response, not intervening, or characterizing symptoms/presentation.

Articles were classified as retrospective or prospective. They were retrospective if they used information from databases or previous documents and prospective if an experimental design or study was completed at the same time as patient enrollment and participation. Articles then listed the statistical analysis methods used.

If information was missing or not readily available the articles were cross referenced in Web of Science, Western (University of Western Ontario) libraries, and google scholar.

Web of Science was most helpful for obtaining information on funding. Western libraries had the most luck with obtaining information on data availability, database usage and funding.

Articles were deemed as not having full text availability if they were not located on Western libraries, PubMed, Web of Science, and Google Scholar.

Data found in the **Supplementary dataset 3**
